# PSOWNNs-CNN: A Computational Radiology for Breast Cancer Diagnosis Improvement Based on Image Processing Using Machine Learning Methods

**DOI:** 10.1155/2022/5667264

**Published:** 2022-05-11

**Authors:** Ashkan Nomani, Yasaman Ansari, Mohammad Hossein Nasirpour, Armin Masoumian, Ehsan Sadeghi Pour, Amin Valizadeh

**Affiliations:** ^1^Department of Electrical and Computer Engineering, Drexel University, Philadelphia, PA, USA; ^2^Department of Computer Engineering, Tehran North Branch, Islamic Azad University, Tehran, Iran; ^3^Department of Medical Genetics, Institute of Medical Biotechnology, National Institute of Genetic Engineering and Biotechnology (NIGEB), Tehran, Iran; ^4^Department of Computer Engineering and Mathematics, Universitat Rovira I Virgili, Tarragona, Spain; ^5^Department of Electrical and Computer Engineering, Kashan Branch, Islamic Azad University, Kashan 8715998151, Iran; ^6^Department of Mechanical Engineering, Ferdowsi University of Mashhad, Mashhad, Iran

## Abstract

Early diagnosis of breast cancer is an important component of breast cancer therapy. A variety of diagnostic platforms can provide valuable information regarding breast cancer patients, including image-based diagnostic techniques. However, breast abnormalities are not always easy to identify. Mammography, ultrasound, and thermography are some of the technologies developed to detect breast cancer. Using image processing and artificial intelligence techniques, the computer enables radiologists to identify chest problems more accurately. The purpose of this article was to review various approaches to detecting breast cancer using artificial intelligence and image processing. The authors present an innovative approach for identifying breast cancer using machine learning methods. Compared to current approaches, such as CNN, our particle swarm optimized wavelet neural network (PSOWNN) method appears to be relatively superior. The use of machine learning methods is clearly beneficial in terms of improved performance, efficiency, and quality of images, which are crucial to the most innovative medical applications. According to a comparison of the process's 905 images to those of other illnesses, 98.6% of the disorders are correctly identified. In summary, PSOWNNs, therefore, have a specificity of 98.8%. Furthermore, PSOWNNs have a precision of 98.6%, which means that, despite the high number of women diagnosed with breast cancer, only 830 (95.2%) are diagnosed. In other words, 95.2% of images are correctly classified. PSOWNNs are more accurate than other machine learning algorithms, SVM, KNN, and CNN.

## 1. Introduction

In the world, breast cancer is one of the leading health problems for women. Breast cancer comes in second place to lung cancer in terms of incidence. According to studies, one out of every nine women will be diagnosed with breast cancer. Approximately 2,088,849 cases of breast cancer were diagnosed globally in 2018 (11.6 percent of all cancer diagnoses) [1, 2]. Breast cancer occurs when there is an overdevelopment of cells in the breast, resulting in lumps or tumors. Malignant tumors tend to penetrate their surroundings more readily and are considered to be cancerous. Benign tumors are less likely to do this [3]. The masses are usually left untreated if they do not cause discomfort to the breast or spread to neighboring tissues. Many types of benign lumps can be found in breasts and prostates, including cysts, fibroadenomas, phyllodes tumors, atypical hyperplasias, and fat necrosis. Tumors can be malignant or invasive. In the absence of early diagnosis and treatment, these lesions spread and damage the surrounding breast tissues, leading to metastatic breast cancer [4, 5]. Metastatic breast cancer occurs when breast tumor cells spread to other organs, such as the liver, brain, bones, or lungs, through the bloodstream or lymphatic system [6]. Breast tissue is mostly made up of glandular (milk-producing) and fat tissues, as well as lobes and ducts. There are numerous types of breast cancer. Ductal and lobular carcinomas are the two most common types of invasive breast cancer [7–9]. In addition to redness, swelling, scaling, and underarm lumps, breast cancer survivors also notice irritation to the skin, fluid leakage, and distorted breasts. The five stages of breast cancer (stages 0 through IV) range from noninvasive malignancy to aggressive breast cancer. There are over 90,000 new cases of these illnesses every year in Asia, and 40,000 people die from them. In part, the growing death rate is due to a lack of knowledge, low education levels, and widespread poverty in diagnosis or consultation with physicians. It may be possible to significantly increase the chance of survival and find more effective treatment options if this condition is diagnosed early. Mammography can reduce mortality by one-third for women over the age of 50 [10, 11].

Because breast cancer cannot be prevented, many manual and image-based exams are useful for identifying and diagnosing it. For early detection of this disease, women are advised to perform a self-exam to become aware of the frequency of bizarre breast anomalies. Breast cancer screenings use a variety of imaging techniques, including X-ray mammography, ultrasound MRI, thermography, and CT scans [12, 13]. Researchers can use these images to examine several breast cancer-related issues. Breast cancer may appear on mammograms as microcalcifications, masses, and architectural deformities, but WSI can also detect abnormalities in the nucleus, epithelium, tubules, stroma, and mitotic activity in breast tissue [14]. In the absence of a tumor, architectural distortion is the hardest abnormality to detect on mammography. Medical breast imaging, such as mammography, is often interpreted differently by expert radiologists. Breast Imaging Reporting and Dated System (BIRADS) was developed by the American College of Radiology to deal with this conflict and radiologists' subjectivity during interpretation and features of breast mammograms, ultrasounds, and magnetic resonance imaging (MRI). Researchers have pioneered the development of artificial neural networks (ANNs) for the detection of breast cancer in recent years. An important aspect of this invention is calculating how many aspects of a diagnostic procedure can be positively affected [15–17].

Additionally, automated detection of breast cancer can mimic the unique behavior of the human brain, making it more effective than manual methods [18, 19]. An ANN cancer detection system mimics the functions of the human brain by approximating and resolving nonlinear and difficult issues, which can be perceived as a mathematical representations-inspired learning process. Further, the predictive accuracy of ANN-based cancer diagnosis is better than that of classic statistical detection approaches due to the latter's reliance on parameter optimization [12, 20]. An ANN-based cancer detection method's performance is also affected by (a) feature selection, (b) learning algorithms and their rates, (c) hidden layer count, (d) multiple nodes in a hidden layer, and (e) initial weights for the factors considered during optimization. When developing ANN-based breast cancer detection systems, feature selection is perhaps the most important factor to consider. ANN-based breast cancer detection techniques rely heavily on feature subsets [21, 22]. Additionally, the input feature subset and the design elements in the ANN-inspired breast cancer diagnostic have a reciprocal relationship. Therefore, the ANN-based process of breast cancer diagnosis must be optimized in terms of feature subset and design parameters [23].

The aim of this research is to reduce uncertainty in order to improve accuracy. Throughout history, uncertainty has always played a role in decision-making, and this is evident by the lack of clarity in the problems. There are times when it is impossible to predict all the parameters of a system, resulting in an incorrect choice. The remainder of this article is organized as follows. The purpose of an artificial neural network is to take in input in the form of a radiological discovery and to generate output in the form of a biopsy. A neural network can be used to identify and predict the risk of breast cancer in masses. In mammography, machine learning methods are used to identify abnormalities by classifying suspicious areas. In the Conclusion, a full assessment of the findings will be presented.

## 2. Related Work

Various deep-learning algorithms have been successfully used to build automated digital models in a variety of applications [24–27]. Using the discrete wavelet transform (DWT) and back-propagation neural networks (BPNN), Beura et al. developed a CAD model based on GLCM features and a BPNN classifier [28]. A KNN classifier was used in conjunction with DWT and GLCM features to develop a CAD model. Based on principal component analysis (PCA) and a support vector machine (SVM) classifier, Liu et al. provided a model that reduced DWT features. DWT and SVM-based CAD models were suggested by Basheer et al. [29]. Linear Discriminant Analysis (LDA) is used in a KNN classifier to extract salient features from a discrete curvelet transform (DCT) model described by [30]. Using lifting wavelet transform features and an extreme learning machine (ELM), Muduli et al. developed a moth flame optimization algorithm to build a hybrid classifier [31]. It produces better classification results with fewer features. Based on support vector machines (SVMs) in particle swarm optimization (PSO), Khan et al. [32] developed an optimized Gabor filter bank CAD model to extract important features and then improve accuracy by using SVM classifiers. The use of ultrasound images for breast cancer classification has also been introduced using machine-learning-based models. A neural network is employed to classify a feature-based model based on autocorrelation coefficients, proposed by Xiao et al. [33]. According to Liu et al. [34], repairing damaged fonts based on style is a better method of repairing damaged fonts. Researchers have found that the font content provided by the research-based CGAN network repair style is comparable to the right font content. Zhou et al. [35] described an efficient blind quality assessment approach for SCIs and NSIs that is based on a dictionary of learnt local and global quality criteria. Li et al. [36] created an artificial intelligence technique that is used for data-enhanced encryption at the IoT's endpoints and intermediate nodes. The technique presented in this article is an AI approach for encrypting data at the endpoints and intermediate nodes of autonomous IoT networks. Yang et al. [37] presented a temporal model for page dissemination in order to reduce the disparity between prediction data from current models and actual code dissemination data. In a study by Eslami et al. [38], attention-based multiscale convolutional neural networks (A+MCNN) were used to efficiently separate distress items from non-distress items in pavement photos. Liao et al. [39] developed an enhanced faster regions with CNN features (R-CNN) technique for semi-supervised SAR target identification that includes a decoding module and a domain-adaptation module named FDDA. Liu et al. [40] developed self-supervised CycleGAN in order to achieve perception consistency in ultrasound images. Sharifi et al. [41] shown how to diagnose tired and untired feet using digital footprint images. According to Zuo et al. [42], deep-learning technologies have improved optical metrology in recent years. He et al. [43] introduced a number of feature selection techniques for reducing the dimensionality of data. Ahmadi et al. [44] developed a new classifier based on wavelet transformation and fuzzy logic. The ROC curve findings show that the given layer is able to accurately segment brain tumors. To predict m6A from mRNA sequences, Zou et al. [45] used word embedding and deep neural networks. Jin et al. [46] developed word embedding and deep neural networks for m6A prediction from mRNA sequences. Yang et al. [47] sought to elucidate the mechanism behind the movement of soy husk polysaccharide (SHP) in the mucus layer triggered by Na+/Ca2+. The findings indicated that Na+ had minimal influence on the viscosity of polysaccharides, but Ca2+ enhanced it. Using a speckle-emphasis-based feature combined with an SVM classifier, Chang et al. [48] suggested a multifeature extraction model that provides the best results. A model that incorporates curvelet, shearlet, contourlet, wavelet, and gray-level cooccurrence matrix (GLCM) features has been proposed by Zhou et al. [49]. For optimal breast cancer detection, Liu et al. [50] proposed an interval-uncertainty-based strategy. Indeterminacy was accounted for using interval analysis. Regardless of the imaging system's alterations, the approach is guaranteed to provide acceptable results. To develop an interval-based Laplacian of Gaussian filter which can be used to simulate intensity uncertainties, the goal was to develop an interval-based Laplacian of Gaussian filter. To demonstrate the method's effectiveness, final findings were applied to the MIAS database and compared with several established methodologies.

A CNN-based method of detecting breast cancer was proposed by Zuluaga et al. [51]. This method was enhanced by BreastNet. Prior to including the image data into the model, the expansion approach was used to establish the image data. An accurate classification system was developed using hypercolumn methodology. To demonstrate the recommended system's increased accuracy, the findings were compared to those of several recent approaches. In histopathology images, Carvalho et al. [52] employed a different method of detecting breast cancer. Phylogenetic diversity indices were used for the construction of models and the categorization of histopathological breast images by the authors. To test its accuracy, the approach was compared to a variety of other recent methodologies. Mahmood et al.'s [53] unique convolutional neural network (ConvNet) used deep learning to identify breast cancer tissues with dramatically lower human error. For identifying mammographic breast masses, the proposed technique obtained a spectacular training accuracy of 0.98, an AUC of 0.99, high sensitivity of 0.99, and test accuracy of 0.97. According to Zhang et al. [54], different identification and detection methods pose both challenges and opportunities, such as amplification of nucleic acids, optical POCT, electrochemistry, lateral flow assays, microfluidics, enzyme-linked immunosorbent assays, and microarrays. Jiang et al. [55] focused on the surface teeth of the entire crown. Robot-assisted trajectory planning is demonstrated to improve efficiency and alleviate pressure associated with manual tooth preparation within the margin of error. Its practicability and validity are demonstrated. Qin et al. [56] suggested a novel monitoring technique for robotic drilling noise based on focused velocity synchronous linear chirplet transforms. Mobasheri et al. [57] reviewed important immunological results in COVID-19 and contemporary reports of autoimmune illnesses related to the condition. According to Ala et al. [58], for solving the appointment scheduling model using a simulation technique, they developed the whale optimization algorithm (WOA), which uses the Pareto archive and the NSGA-II algorithm. An adaptive secondary sampling method based on machine learning for multiphase drive systems is proposed by Liu et al. [59]. Zheng et al. [60] recommended image classification as the research goal for examining how metalearning rapidly acquires knowledge from a limited number of sample photos. In an article, Liu et al. [61] developed an image stitching algorithm based on feature point pair purification. Kaur et al. [62] have used a deep convolutional neural network (DCNN) and fuzzy support vector machines; they have developed two-class and three-class models for breast cancer detection and classification. Mammogram images from DDSM and curated breast imaging subsets DDSM (CBIS-DDSM) were used to create the models. Our system was tested for accuracy, sensitivity, AUC, F1-score, and confusion matrix. For the DCNN and fuzzy SVM, the accuracy of the 3-class model was 81.43 percent. With a 2-layer serial DCNN with fuzzy SVM, the first layer achieved accuracies of 99.61 percent and 100.00 percent, respectively, in binary prediction.  Table 1 shows the summary of related work.

To get high-frequency and low-frequency pictures, Li et al. [69] employed a wavelet for multiscale decomposition of the source and fusion images. This article employed a deep convolutional neural network to learn the direct mapping between the high-frequency and low-frequency pictures of the source and fusion images in order to get clearer and more comprehensive fusion images. The experimental findings demonstrated that the approach suggested in this research may produce a good fusion picture that is superior in terms of both visual and objective assessments to that produced by certain sophisticated image fusion algorithms. In their study, Zhang et al. [70] used the Gaussian pyramid to improve the basic ORB-oriented approach. Based on the experimental results, we have demonstrated that the pyramid sphere method is invariant, resilient to scale and rotation changes, and has a high degree of registration accuracy. Additionally, the stitching speed is around ten times to that of SIFT. Shan et al. [71] employed a two-dimensional three-dimensional multimode medical image registration technique based on normalized cross-correlation. The results demonstrate that a multiresolution technique enhances registration accuracy and efficiency by compensating for the normalized cross-correlation algorithm's inefficiency. Xu et al. [72] suggested a technique for segmenting and categorizing tongue pictures using an MTL algorithm. The experimental findings demonstrate that our combined strategy outperforms currently available tongue characterisation techniques. Ahmadi et al. [73] used a CNN to segment tumors associated with seven different types of brain disorders: glioma, meningioma, Alzheimer's, Alzheimer's plus, Pick, sarcoma, and Huntington's. Sadeghipour et al. [74] developed a new method, combining the firefly algorithm and an intelligent system, to detect breast cancer. Researchers Zhang et al. [75] explored a way to query clinical pathways in E-healthcare systems while preserving privacy. According to Sadeghipour et al. [76], a new designed system was developed for diagnosing diabetes using the XCSLA system. Ahmadi et al. [77] introduced a technique called QAIS-DSNN for segmenting and distinguishing brain malignancies from MRI images. The simulation results obtained using the BraTS2018 dataset indicate that the suggested technique is 98.21 percent accurate. Chen et al. [78] developed a model of label limited convolutional factor analysis (LCCFA) that combines factor analysis (FA), convolutional operations, and supervised learning. Our technique outperforms other relevant models in terms of classification accuracy for small samples on multiple benchmark datasets and measured radar high-resolution range profile data. Rezaei et al. [79] created a data-driven technique for segmenting hand parts on depth maps that does not need additional work to get segmentation labels. Sadeghipour et al. [80] created a clinical system for diagnosing obstructive sleep apnea with the XCSR Classifier's assistance. Dorosti et al. [81] developed a generic model to determine the link between several characteristics in a GC tumor's location and size. Abadi et al. [82] suggested an unique hybrid salp swarm algorithm and genetic algorithm (HSSAGA) model for scheduling and designating nurses. The proposed test function algorithm's results indicate that it outperforms state-of-the-art techniques. Zhou and Arandian [83] proposed a computer-aided technique for skin cancer detection. A mix of deep learning and the Wildebeest Herd Optimization Algorithm was used to create the approach. The first characteristics are extracted using an Inception convolutional neural network. Following that, the WHO method was used to choose the relevant characteristics in order to reduce the analysis time complexity. Finally, the entire diagnostic system was compared to other ways in order to determine its efficacy in comparison to the methods evaluated. Davoudi et al. [84] examined the effect of statins on the severity of COVID-19 infection in patients who had been taking statins prior to infection. Hassantabar et al. [85] examined the effect of statins on the severity of COVID-19 infection in patients who had been taking statins prior to infection. Yu et al. [86] used differential expression analysis to combine the biological relevance of genes from gene regulatory networks, resulting in weighted differentially expressed genes for breast cancer detection. The binary classifier was capable of making a decent prediction for an unknown sample, and the testing results confirmed the efficacy of our suggested methods. A convolutional neural network based on an artificial fish school technique was suggested by Thilagaraj et al. [87]. The breast cancer image dataset comes from archives of cancer imaging. The breast cancer picture was filtered with the use of a Wiener filter for classification in the preprocessing phase of classification. By determining the number of epochs and training pictures for the deep CNN, the optimization approach assisted in lowering the error rate and increasing performance efficiency.

## 3. Breast Cancer Detection and Diagnosis

Prediction and treatment of breast cancer using computers are largely based on intermediate procedures such as segmenting (identifying breast lesions), identifying features, and finally categorizing areas found into classes. It is possible to detect breast lesions by either defining a suspicious region pixel by pixel in a breast image or by creating a bounding box around the suspicious area. Cancer could be detected by processing whole breast images instead of removing worrisome spots and then categorizing them, which would incur an additional charge. To classify the lesions under investigation, features are extracted from the ROI or the whole image. A classification algorithm (ML or DL) uses these features to classify the samples.

### 3.1. Features Learning

There are many aspects of this work which are depicted through the images. Segmenting and classifying images require knowledge of the most informative and accurate features. A large and complicated set of features are extracted due to the discrepancy between benign and malignant lesions. As a result, selecting the right set of features is crucial, since having too many features can degrade the classifier's performance and increase its complexity. To segment and classify breast lesions, numerous kinds of handmade features, such as texture, size, shape, intensity, and margins, were previously obtained from breast images. [12].

**Figure 1:**
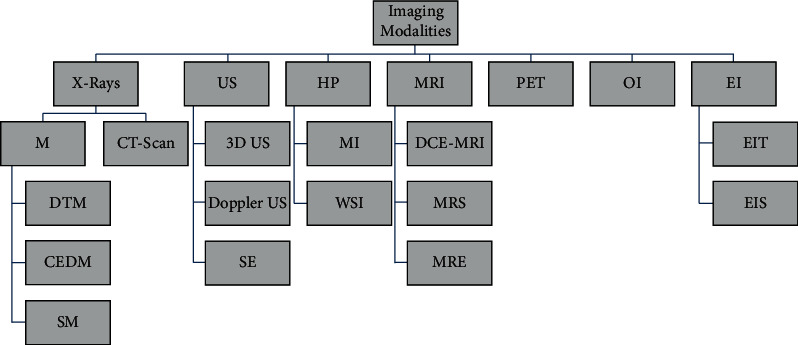
Modalities of imaging.

As a result, deep learning has considerably improved the whole feature extraction process, thereby improving the performance of the following stages (e.g., detection and classification). Hence, deep features derived from a convolutional network trained on a large dataset can perform discriminating tasks far better than conventional approaches based on hand-engineered features or typical machine learning methods (see Figure 1).

## 4. Proposed Method

The flow chart of the structure of CNN used can be seen in Section 4.7. Our approach to improving Shafer's hypothesis was a combination of approaches as you can see in the image. Machine learning and neural networks are used to classify and diagnose tumors. For this purpose, CNN deep neural networks are individually trained and tested. This article discusses two strategies for feature extraction. CNN uses deep features for feature extraction. With gray-level cooccurrence matrix features retrieved from the image, an artificial neural network is trained. In the subsequent stages, a classifier is used to determine the probability of each class.

### 4.1. Dataset

This study used Mini-MIAS Screening mammography data as input images. Data would be gathered directly from hospitals and physicians, as well as from public sources. Data would be publicly available. Image resolution is 256 × 256 pixels in PNG format. This is an example of an image. 1824 images are used for analysis and simulation: 80% for training and 20% for validation.

### 4.2. Analyzing Outliers and Reducing Dimensions

To reduce the dimensionality of the data, Principal Component Analysis (PCA) was used. To determine the appropriate number of principal components, several machine learning models were fitted repeatedly to the modified data. In order to evaluate the effects of dimensionality reduction on prediction accuracy, a predefined number of principal component analyses (PCA) were conducted before training all models using the Classifying Learner application in the Statistics and Machine Learning Toolbox. Principal component analysis of the data was used to identify the “base” model. In order to determine the appropriate number of principal components for the modified data, machine learning models were fitted repeatedly. The PCA was performed independently on benign and malignant tumors to remove outliers.

### 4.3. ROI Preprocessing

**Figure 2:**
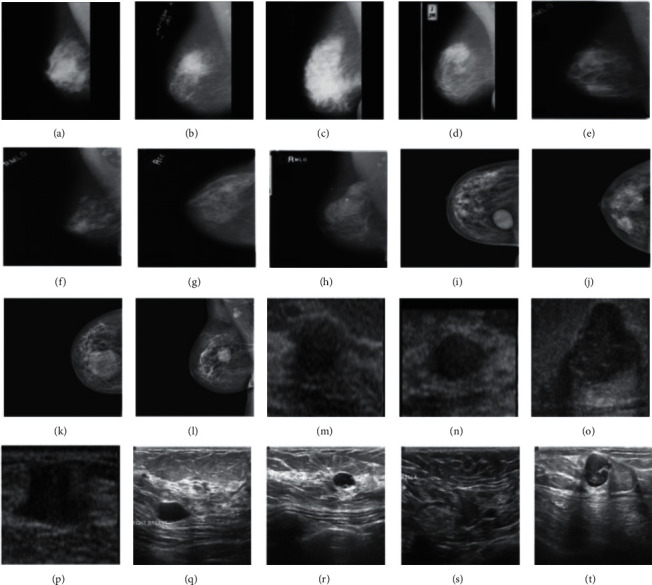
Sample benign images from different datasets like MIAS ((a) and (b)), DDSM ((e) and (f)), INbreast ((i) and (j)), BUS-1 ((m) and (n)), and BUS-2 ((q) and (r)). Sample malignant images from different datasets like MIAS ((c) and (d)), DDSM, ((g) and (g)), INbreast ((k) and (l)), BUS-1 ((o) and (p)), and BUS-2 ((s) and (t)).

A variety of undesirable visual features, noise, and artifacts can be seen in the mammography images in the database, resulting in a low-quality image that will inevitably lower the accuracy of classification. As a result of manually cropping each image in the MIAS, we obtained the Region-of-Interest (ROI) encompassed by the suspected anomaly. To crop the image to size, the radiologist provided the center and radius parameters in the dataset (Figure 1). The ROIs that were retrieved are shown in Figure 2. Size has been assigned to the INbreast, BUS-1, and BUS-2 full images due to a lack of ground truth data. Figure 2 shows the MIAS, DDSM, INbreast, BUS-1, and BUS-2 datasets [69].

### 4.4. Feature Extraction

To minimize the number of resources required for an accurate display of a large amount of data, feature extraction is used. When collecting complex data, the number of variables being examined is one of the most significant challenges. Using the instructional example, a large number of variables requires a large amount of memory and storage. To solve problems requiring high precision, feature extraction is a term that refers to a wide range of methodologies for gathering data. The idea behind image analysis is to design a unique method for representing the fundamental components of an image distinctively. The fractal approach was used to generate gray area vectors for feature vectors. Based on the light intensity of the defined locations relative to someone in the image, statistical analysis is used to create the image features of the confidence interval for the identified chemicals. In each combination of intensity points (pixels), the statistics are affected by the frequency of these points. We extract the feature using the GLCM model in this study. The feature selection technique was used to reduce the dimensions and identify additional critical qualities that might adequately separate the various systems in terms of their capacity to interact with large amounts of input data [48]. The GLCM approach was combined with covariance analysis to extract eigenvalues and reduce the size of the image. The fractal approach requires identical input images, and each image is considered a two-dimensional matrix and a single vector. Images must be grayscale and of a certain resolution. As a result of matrix reshaping, each image becomes a column vector, and each image is extracted from an MN matrix, where N is the number of pixels in each image, and M is the total number of images. It is necessary to compute the average image for each original image in order to establish the normal distribution. The covariance matrix is then calculated as well as the eigenvalues and eigenvectors. Fractal systems use the following mechanism: M is the number of training images, Fi is the mean of the images, and li represents each image in the Ti array, beginning with M images, each of which has NN pixels.

### 4.5. Concept of Convolutional Neural Network (CNN)

**Figure 3:**
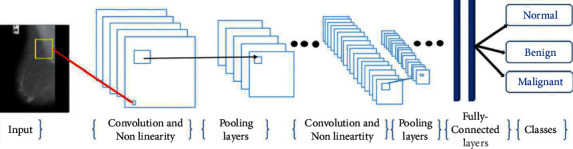
A description of a CNN architecture.

Convolutional neural network (CNN) is a significant technique from the deep learning field. A CNN typically includes principal layers of the convolution, the Maxpooling, the fully connected layer, and the other layers executing various features. There are two phases for the preparation of each system, the forward progression and backward progression. Firstly, the data moves from the input layer to the hidden layer and then to the output layer. In the backpropagation algorithm, the input image is doing the feeding process to the network in the first step. Once the output is achieved, the error value is evaluated. This value is then brought back to the network together with updating the network weight and along with the cost functions diagram (see Figure 3). CNN consists of different types of hidden sublayers as discussed below.

*Convolutional Layer*. It is the principle of the convolution network. This layer's output is represented as a 3D neuron matrix. The CNN network requires multiple kernels in certain layers to transform both the input image and the core function maps. The convolution process has three key advantages:

- The weight-sharing method decreases the number of features in each function diagram.
- The connection between adjacent pixels is known through a local connection.
- It induces changes in the position of the target to create equilibrium.

*Activation Functions*. In particular, activation functions are used in neural networks to achieve the desired output. Neural networks can use various activation functions; the most significant of which are Tanh activation functions and Sigmoid. The sigmoid function transforms input data (−∞–+∞) to values from 0 to 1. Tanh provides production value 1 to −1 interval. One of the other activation functions is the ReLU function which has been introduced in recent years. ReLU is a function of activation *g* extended to all components. It aims to present the network with nonlinear behavior. This functionality contains all pixels in the image and converts all negative points to 0.

  Max pooling: There are several consequences of the use of Maxpooling in CNN. The use of it helps CNN to define the target with only small modifications to the matrix at first. Second, it helps CNN to recognize features in the huge size of the image. CNN Maxpooling is used to sum up the functions during the sampling process subtraction and so can be gotten into deep steps of CNN. Then we need to begin pooling to get what we have to retain this information. Among the most common forms of pooling are Max and Average.
  Data augmentation: Preprocessing and data enhancement are some of the most often overlooked issues. However, these tasks are often unnecessary. You should always check whether your task needs to be preprocessed before running any data processing.

### 4.6. Classifier's Performance Analysis Metrics

**Figure 4:**
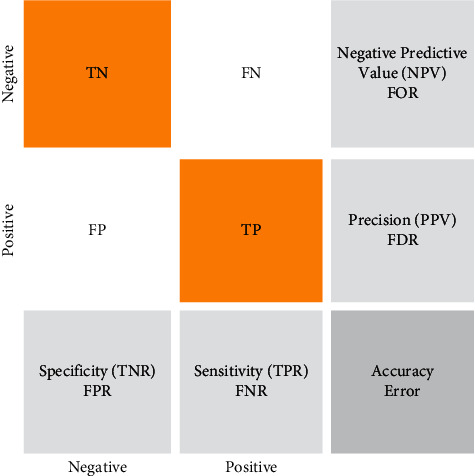
The confusion matrix.

By comparing the true positive rate (TPR) to the false positive rate (FPR), the receiver operating characteristic (ROC) curve is established. In machine learning, TPR is sometimes referred to as recall or probability of detection. The FPR and TPR have disappeared from the left side of the ROC (see Figure 4). There are a lot of meaningful test findings on the threshold line. Start with the most recent findings, which represent the most meaningful test results. The consistency with which a measure categorizes knowledge into these two categories is quantifiable and informative. The study described here emphasizes the importance of specificity over recall (also known as responsiveness or TPR) because low precision leaves patients with no need for therapeutic intervention. On the other hand, recall should not be neglected as a false positive result could lead to unnecessarily treating the individual. Evaluation of the models included recall and specificity, fivefold cross-validated accuracy, F1-score, and Matthew's correlation coefficient (MCC). The accuracy is determined by the number of correctly detected observational events (both benign and malignant tumors); F1 represents the harmonic mean of precision and recall and signifies the model's ability to predict. In other words, precision refers to the proportion of accurately detected malignancies in the anticipated set to the total number of malignancies. Each of recall, specificity, accuracy, and precision has a value between 0 and 1. The MCC was evaluated as a metric for assessing classification accuracy, since it takes into account both true negatives as well as true positives, false positives, and false negatives, and when classes are imbalanced; it can measure classification accuracy more accurately. The most commonly used binary classification evaluation measures are accuracy and F1-score when assessing models' performance on unbalanced datasets. It is only when a model predicts accurately in all four confusion matrix categories (true positives, false negatives, and false positives) that it is considered high in the MCC for binary classification. MCC provides more accurate estimates of classification accuracy when applied to the WBCD dataset. There is a perfect prediction on an MCC score of 1 if predictions and observations do not agree. The formulas for recall, specificity, accuracy, precision, and F1-score are listed as follows.

(1) $$\begin{array}{c} \text{Recall }=\frac{TP}{TP+FN},\\ \text{Specificity }=\frac{TN}{TN+FP},\\ \text{Accuracy }=\frac{TP+TN}{TP+TN+FP+FN},\\ \text{Precision }=\frac{TP}{TP+FP},\\ F_{1}=\frac{2TP}{2TP+FN+FN},\\ MCC=\frac{TP\times TN-FP\times FN}{\sqrt{\left(TP+FP\right)\left(TP+FN\right)\left(TN+FP\right)\left(TN+FN\right)}}. \end{array}$$

A true positive, a true negative, a false positive, and a false negative, respectively, are represented in the above equations. Mixture-effect models were run in JMP to determine the effects of dimensionality reduction, outlier analysis, or combinations of both on cross-validated accuracy.

### 4.7. CNN Neural Network Structure

**Figure 5:**
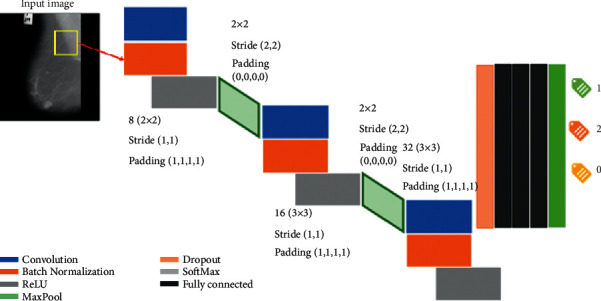
The structure of CNN.

CNN has been a major factor in deep learning's recent success. The convolutional layers in this neural network are completely linked to the top layer. Additionally, the weights and layers are combined in this approach. The performance of this deep neural network was better than previous deep neural network designs. Furthermore, deep-feed neural networks are simpler to train. They are useful because they have a limited number of estimated parameters. Convolutional neural networks consist of three primary layers: the convolutional layer, the pooling layer, and the fully connected layer, each of which performs a unique function. There are two steps to training convolutional neural networks: feedforward and backpropagation. After feeding the input image into the neural network, the network output is calculated, which is used to adjust the network training parameters; after calculating the output, the results are used to calculate error rates for the network. Starting with the error value computed in the previous phase, backpropagation begins. The gradients of each parameter are computed using the chain rule, and each parameter varies in response to its influence on the network's error. Afterward, the feedforward phase begins. After a certain number of iterations, the training concludes. Our structure of the convolutional network is illustrated in Figure 5 and Table 2. There are twenty layers in the structure of CNN.

## 5. Results and Discussion

**Figure 6:**
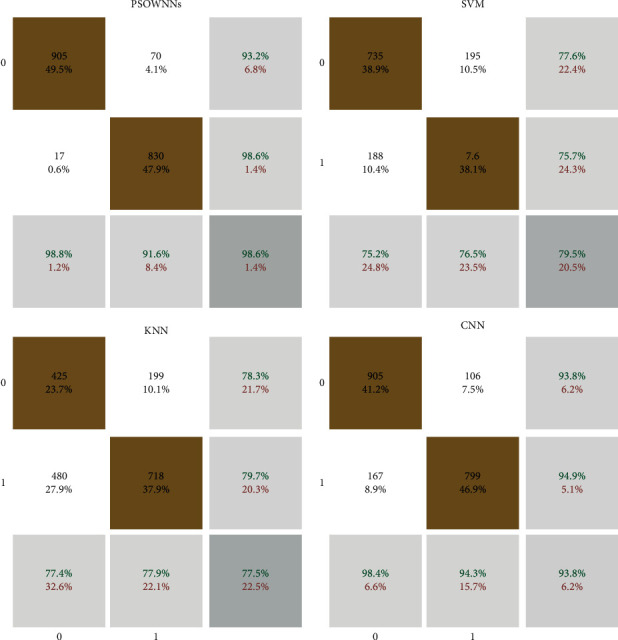
The confusion matrix of the deep learning methods used for breast cancer diagnosis.

To evaluate, we have divided the test data into two categories, benign and malignant. The evaluation was performed on 64 samples from the benign class and 51 representatives from the malignant class from the MIAS dataset. The test data related to the benign class and the probability of belonging are considered first. The test data associated with the malignant class and its chance are considered in the second category. We now discuss the ROC, confusion matrix, and the comparison diagrams of the two classes.  Figure 6 shows the ROC of the MLP with GLCM features, as well as CNN for the benign and malignant class.

The techniques are also included in each class of 905 matrices with 22 features.  Figure 6 shows the classification results in a confusion matrix. Hence, in the confusion matrix in Figure 6, green cells indicate real metrics, while white cells indicate false metrics. In findings, out of 905 images of breast cancer, 830 (47.2 percent) are diagnosed correctly. Despite this, 70 (4.1 percent of all images) are misdiagnosed. So PSOWNNs techniques have a sensitivity of 91.6%. In other words, 91.6 percent of individuals with breast cancer are identified correctly. Moreover, 905 images with different disorders were correctly diagnosed in 98.8 percent of the cases. PSOWNNs have a specificity of 98.8%. Additionally, PSOWNNs have an accuracy of 98.6%, which means 830 patients are found to have breast cancer, they (98.6%) are correctly diagnosed. The accuracy of classifiers is another significant parameter. PSOWNNs have an accuracy of 95.2%. That means that 95.2% of the images are correctly diagnosed. In comparison with other machine learning techniques, PSOWNNs are more accurate than SVM, KNN, and CNN. As neural networks do, CNNs train to build output maps by removing more complex, high-level functions. Input function maps are combined with convolutional kernels. CNN exploits the fact that a feature is the same in the receptive field irrespective of its location if a function converts. The results show that CNN can acquire more useful functionality than techniques that do not take this into account. As a result of this assumption, weight sharing is used to decrease the number of components.

The used methods are trained by gradient descent. Hierarchical characteristics are optimized for the task at hand because each layer feeds into the previous one. A real-valued vector is typically needed for SVM and other methods. By contrast, CNN is often taught from beginning to end, ensuring that it reacts appropriately to the challenge it is attempting to resolve. In SVMs, KNNs, and CNNs, PSOWNNs are used as trainable attribute detectors. Since SVMs are still extensively used, different machine learning algorithms should complement each other. Consequently, this article uses machine learning to identify breast cancer based on GLCM traits. CNN's confusion matrix is depicted in Figure 5. 905 breast cancer patients were accurately diagnosed by 799 (94.9 percent) according to the matrix. However, 106 images were misdiagnosed. On the other hand, all patients with additional disorders or negative test results are identified. The CNN approaches have a sensitivity of 94.3 percent and a specificity of 93.8 percent, respectively. They also have a 93.4 percent accuracy rate. Breast cancer is a true positive in all patients with this identification. CNN methodologies, therefore, are 95.9% accurate.

**Figure 7:**
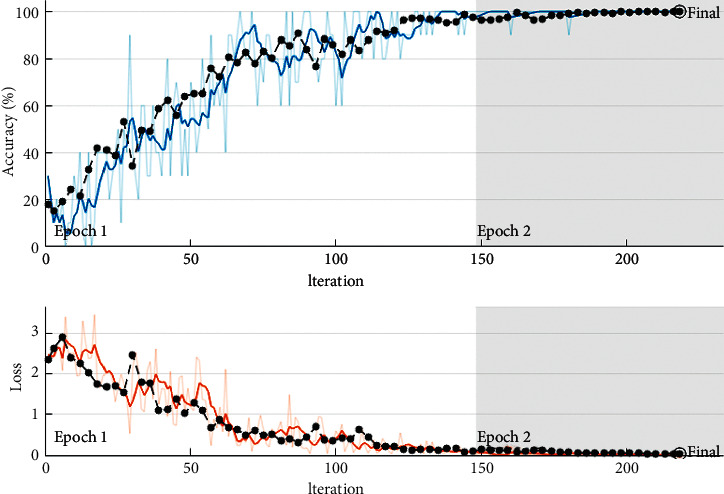
The training process of the CNN approach.

**Figure 8:**
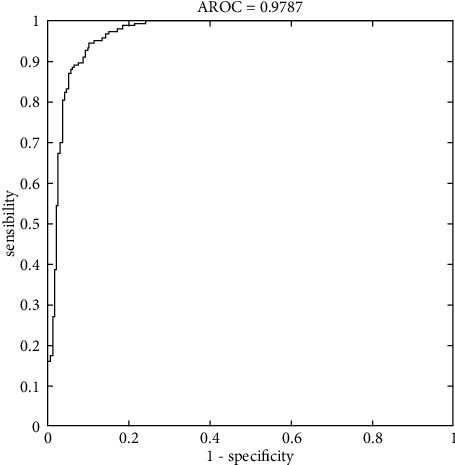
ROC region for the breast tumor segmentation in the MIAS dataset.

In terms of classification, it is preferable if the fall-out and sensitivity have lower and higher values, respectively. CNN has, therefore, a greater sensitivity than other methods. Meanwhile, KNN demonstrated that machine learning algorithms are less sensitive than CNN (see Figure 7). In Figure 8, the ROC curve is displayed to compare machine learning algorithms. Fall-out is represented by the horizontal axis of the ROC curve, while sensitivity is represented by the vertical axis.

Based on the performance analysis metrics results shown in Table 3, the higher values belong to the CNN technique. ROC curve value is an essential metric for classifiers. For CNN methods, it is 99.97. Consequently, the highest accuracy belongs to CNN, KNN, LDA, SVM, and NB, respectively.

## 6. Discussions

The improved SVM achieves the lowest score for all criteria on the Mini-MIAS mammography dataset. ANNs are also derived from KNNs and CNNs, but their major drawbacks are overfitting, hence the need for more training data, as well as their inability to extract features. Among the most sensitive and specific structures are machine learning methods. As a result of their less sensitivity to variance in input samples, convolutional neural networks outperform CNNs and PSOWNNs. Results show that the PSOWNNs have the best classification performance and lowest error rate. Based on the Mini-MIAS datasets, Figure 8 shows the receiver operating characteristics (ROC) area for the used structure. ROC analysis involves analyzing classification jobs at different threshold values. A statistical model's accuracy and, more broadly, the performance of a system can be evaluated based on this evaluation.

**Figure 9:**
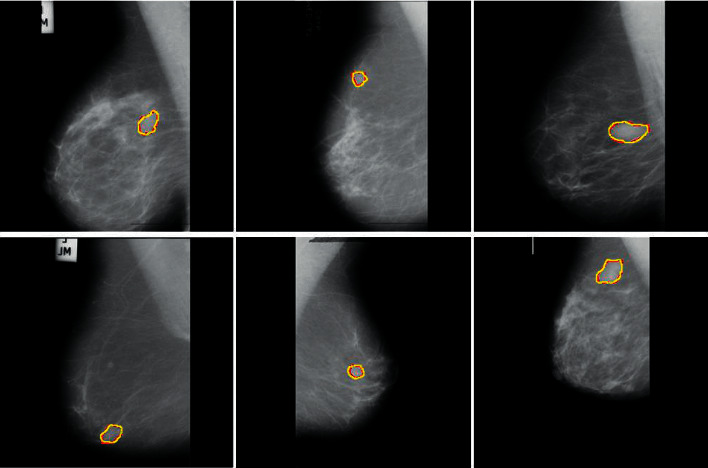
CNN for Mini-MIAS dataset was used to visually assess breast tumor segmentation.

From the Mini-MIAS mammography dataset shown in Figure 9, you can see a visual representation of how the technique is used to segment different tissues and tumors.

## 7. Conclusion

A complicated illness, breast cancer is recognized as the second leading cause of cancer-associated mortality in women. A growing body of data suggests that many factors (e.g., genetics and environment) might play a role in breast cancer onset and development. Image-based diagnostic methods are among the many diagnostic platforms that can provide valuable information on breast cancer patients. Unfortunately, it is not always easy to identify breast abnormalities. Various technologies have been developed to screen for breast cancer, such as mammography, ultrasound, and thermography. Utilizing image processing and artificial intelligence (AI) techniques, the computer helps radiologists identify chest problems more effectively. This article evaluated many approaches to detecting breast cancer using AI and image processing. Using machine learning methods for identifying breast cancer, the authors present an innovative approach. Based on the support value on a neural network, the suggested method differs from previous techniques. A normalizing technique has been implemented to benefit the image in terms of performance, efficiency, and quality. According to our experiments, PSOWNNs are relatively superior to current approaches such as CNN. There is no doubt that machine learning methods are beneficial in terms of performance, efficiency, and quality of images, which are vital to the newest medical applications. Based on the results, PSOWNNs approaches are 91.6% sensitive. That is, 91.6 percent of breast cancer patients are detected correctly. A further comparison of the process 905 image with those of other illnesses reveals that 98.6 percent of the disorders are correctly diagnosed. Therefore, PSOWNNs have a specificity of 98.8%. Furthermore, PSOWNNs have a precision of 98.6 percent, which means 830 people are seen to have breast cancer, but only 830 (95.2 percent) are diagnosed. In other words, 95.2% of images are correctly classified. PSOWNNs are more accurate than other machine learning algorithms, SVM, KNN, and CNN.

## Figures and Tables

**Table 1:** Summary of related work.

Author	Year	Type	Network	Result	Advantages	Disadvantages

Dong et al. [22]	2022	Breast cancer diagnosis and classification	Random forest and regression tree	The application of machine learning techniques like CART and random forests coupled with geographical methodologies provides a viable alternative for future inequalities studies	(i) Low complexity (ii) High accuracy	(i) Possible overfitting (ii) Used classic feature extraction (iii) Lowest robustness

Guha et al. [19]	2022	Breast cancer risk factors	SEER-Medicare analysis	The incidence of AF in women after a breast cancer diagnosis is much higher. AF is strongly linked to a higher stage of breast cancer upon diagnosis. Women newly diagnosed with breast cancer who develop AF suffer an increased risk of cardiovascular death but not breast-cancer-related death	(i) Ability of risk assessment (ii) Technical assessment	(i) Needs feature extraction (ii) Unable to diagnose the patient

Chamieh et al. [63]	2022	Breast cancer diagnosis using fine-needle aspiration cytology	Begg and Greenes method	Irrespective of the recommended technique, the FNAC test's specificity was always greater than its sensitivity. For all approaches, the probability ratios were positive. Both positive and negative yields were high, demonstrating the test's exact discriminating qualities.	(i) Technical assessment method (ii) Low complexity	(i) Unable to diagnose illness type (ii) Limited dataset

Thangarajan et al. [64]	2022	Breast cancer biomarkers validated in plasma	BC gene expression profiling	Methylation status of SOSTDC1, DACT2, and WIF1 can distinguish BC from benign and control conditions with 100% sensitivity and 91% specificity. Therefore, SOSTDC1, DACT2, and WIF1 may be used as a supplemental diagnostic tool to distinguish noninvasive and invasive breast cancer from benign breast conditions and healthy individuals	(i) Using biomarkers instead of mathematical features	(i) Lower sensitivity (ii) Unable to diagnose illness type

Chakravarthy et al. [65]	2022	Diagnosis of breast cancer	Multideep CNN	By fuzzing deep features for both datasets (97.93 percent for MIAS and 96.646 percent for INbreast), we achieved the highest classification accuracy among state-of-the-art frameworks. When the PCA was applied to combined deep features, classification performance did not improve, but execution time was shorter, resulting in a lower computing cost	(i) Low complexity (ii) High accuracy (iii) Technical assessment method	(i) Possible overfitting (ii) Needs feature extraction (iii) Lower sensitivity (iv) Lowest robustness
Wang et al. [66]	2022	Metastasis of breast cancer axillary lymph nodes forecasting	CNN	The T2WI sequence outperformed the other three sequences in the testing set, with accuracy and AUC of 0.933/0.989. In comparison with T1WI, which has accuracy and AUC of 0.691/0.806, the increase is substantial	(i) Ability of risk assessment (ii) Technical assessment	(i) Unable to diagnose the patient (ii) Used classic feature extraction (iii) Limited dataset (iv) Lower sensitivity

Melekoodappattu et al. [67]	2022	Breast cancer detection	CNN and texture feature-based approach	Using our ensemble method, we measured 97.8% specificity and 98.6% accuracy for MIAS and 98.3% and 97.9% for DDSM. Experimental data indicate that the combination strategy enhances measurement metrics independently for each phase	(i) Low complexity (ii) High accuracy	(i) Unable to diagnose illness type

Gonçalves et al. [68]	2022	Breast cancer detection	CNNs	VGG-16 produced F1-scores greater than 0.90 for all three networks, an increase from 0.66 to 0.92. Furthermore, compared to earlier research, we were able to improve the F1-score of ResNet-50 from 0.83 to 0.90	(i) Comparative study (ii) Used high-rank methods	(i) Unable to diagnose illness type (ii) High complexity

**Table 2:** The presented CNN architecture layers.

Layer	Name	Description
1	Image input	256 × 256 × 1 images with “zero center” normalization
2	Convolution	8 (3 × 3) convolutions with stride [1 1] and padding “same”
3	Batch normalization	Normalization
4	ReLU	Rectifier
5	Pooling	2 × 2 max pooling with stride [2 2] and padding [0 0 0 0]
6	Convolution	16 (3 × 3) convolutions with stride [1 1] and padding “same”
7	Batch normalization	Normalization
8	ReLU	Rectifier
9	Pooling	2 × 2 max pooling with stride [2 2] and padding [0 0 0 0]
10	Convolution	32 (3 × 3) convolutions with stride [1 1] and padding “same”
11	Batch normalization	Normalization
12	ReLU	Rectifier
13	Fully connected	7 fully connected layers
14	SoftMax	SoftMax
15	Classification output	Cross entropy

**Table 3:** The comparison between the presented methods.

Methods	Sensitivity (%)	Specificity (%)	Precision (%)	AUC	Accuracy (%)
KNN	77.9	77.4	79.7	79.43	77.5
SVM	78.5	75.2	75.7	78.54	79.5
PSOWNNs	91.6	98.8	98.6	95.43	95.2
CNN	94.3	93.4	94.9	93.65	93.8

## Data Availability

Data are available and can be provided over the emails querying directly to the corresponding author (amin.valizadeh@mail.um.ac.ir).

## Nomenclature

ANN: Artificial neural network
BIRADS: Breast Imaging Reporting and Dated System
CNN: Convolutional neural network
DCNN: Deep convolutional neural network
DL: Deep learning
FN: False negative
FP: False positive
FPR: False positive rate
GLCM: Gray-level cooccurrence matrix
KNN: k-nearest neighbor
LDA: Linear discrimination analysis
MCC: Matthew's correlation coefficient
ML: Machine learning
NB: Naïve bayes
PCA: Principal component analysis
PSOWNN: Particle swarm optimized wavelet neural network
ReLU: Rectified linear unit
ROC: Receiver operating characteristic
ROI: Region-of-Interest
SVM: Support vector machine
TN: True negative
TP: True positive
TPR: True positive rate.
